# Three-fold rotational defects in two-dimensional transition metal dichalcogenides

**DOI:** 10.1038/ncomms7736

**Published:** 2015-04-02

**Authors:** Yung-Chang Lin, Torbjörn Björkman, Hannu-Pekka Komsa, Po-Yuan Teng, Chao-Hui Yeh, Fei-Sheng Huang, Kuan-Hung Lin, Joanna Jadczak, Ying-Sheng Huang, Po-Wen Chiu, Arkady V. Krasheninnikov, Kazu Suenaga

**Affiliations:** 1 National Institute of Advanced Industrial Science and Technology (AIST), Tsukuba 305–8565, Japan; 2 COMP/Department of Applied Physics, Aalto University, P.O. Box 11100, FI-00076 Aalto, Finland; 3 Department of Electronic Engineering, National Tsing Hua University, Hsinchu 30013, Taiwan; 4 Department of Electronic Engineering, National Taiwan University of Science and Technology, Taipei 10607, Taiwan; 5 Institute of Physics, Wrocław University of Technology, Wyb. Wyspiańskiego 27, 50-370 Wrocław, Poland; 6 Department of Applied Physics, Aalto University, P.O. Box 11100, FI-00076 Aalto, Finland

## Abstract

As defects frequently govern the properties of crystalline solids, the precise microscopic knowledge of defect atomic structure is of fundamental importance. We report a new class of point defects in single-layer transition metal dichalcogenides that can be created through 60° rotations of metal–chalcogen bonds in the trigonal prismatic lattice, with the simplest among them being a three-fold symmetric trefoil-like defect. The defects, which are inherently related to the crystal symmetry of transition metal dichalcogenides, can expand through sequential bond rotations, as evident from *in situ* scanning transmission electron microscopy experiments, and eventually form larger linear defects consisting of aligned 8–5–5–8 membered rings. First-principles calculations provide insights into the evolution of rotational defects and show that they give rise to p-type doping and local magnetic moments, but weakly affect mechanical characteristics of transition metal dichalcogenides. Thus, controllable introduction of rotational defects can be used to engineer the properties of these materials.

## Abstract (web summary)

Defects in a crystalline solid can have a dramatic effect on the material’s properties. Here, the authors demonstrate a class of defects in two-dimensional transition metal dichalcogenides mediated by chalcogen vacancies and inherently related to the crystal symmetry

## Graphical abstract

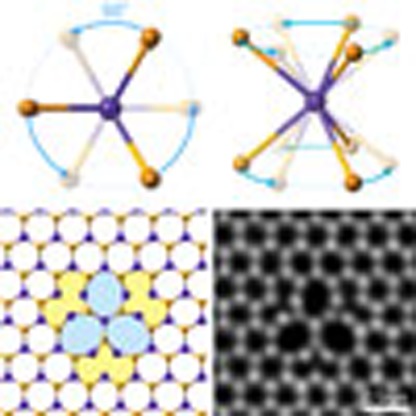

Point and line defects strongly influence electronic, optical, thermal and mechanical properties of solids, either with overall detrimental (for example, defect-induced embrittlement of reactor steels) or beneficial (doping of semiconductors) effect on the materials characteristics, calling on careful investigations of their atomic structure. Aberration-corrected transmission electron microscopy (TEM) made it possible to study the structure and behaviour of defects on atomic scale in real time, and has provided many insights into defect dynamics. In sp^2^-hybridized hexagonal carbon systems, for example, carbon nanotubes^1^ or graphene^2^,^3^, Stone–Wales transformations^4^ representing rotations of C–C bonds by 90° (Fig. 1a,b) have been observed. Such transformations can be regarded as an elementary step that not only gives rise to topological defects^5^,^6^, but are also responsible for plastic deformation^7^,^8^, rippling^9^ and grain boundary motion^10^.

In two-dimensional (2D) materials with trigonal symmetry, for example, h-BN^11^,^12^,^13^,^14^ or transition metal dichalcogenides (TMDs)^15^,^16^,^17^,^18^,^19^,^20^, point and line defects have been observed, but Stone–Wales rotational defects are not expected due to the polar nature of chemical bonds in such systems. On the other hand, a 60° rotation of three bonds centred on a metal atom, as schematically shown in Fig. 1e–g, would preserve the heteroatomic nature of bonding and the trigonal lattice symmetry, resulting in the formation a ‘trefoil’-shaped defect (Fig. 1h). This gives rise to the fundamental question: can such transformations occur in stoichiometric or atom-deficient 2D systems with trigonal symmetry, and if yes, what kind of defects they would produce and how would these defects affect the properties of the material.

In the following, we present observations of such rotational defects. By combing scanning TEM (STEM) experiments with first-principles calculations, we show that such rotational defects exist in chalcogen-deficient TMDs and that the trefoil defect (Fig. 1j) is the simplest example in a series of bond rotation-mediated transformations in the TMDs. By sequential rotations of metal–chalcogen (M–X) bonds, the rotational defects can expand in size and also migrate in the lattice, or form one-dimensional domain boundaries. The formation and evolution of the defects are mediated by chalcogen vacancies induced by the electron-beam irradiation.

## Results

### Atomic structure of rotational defects in TMDs

**Figure 1:**
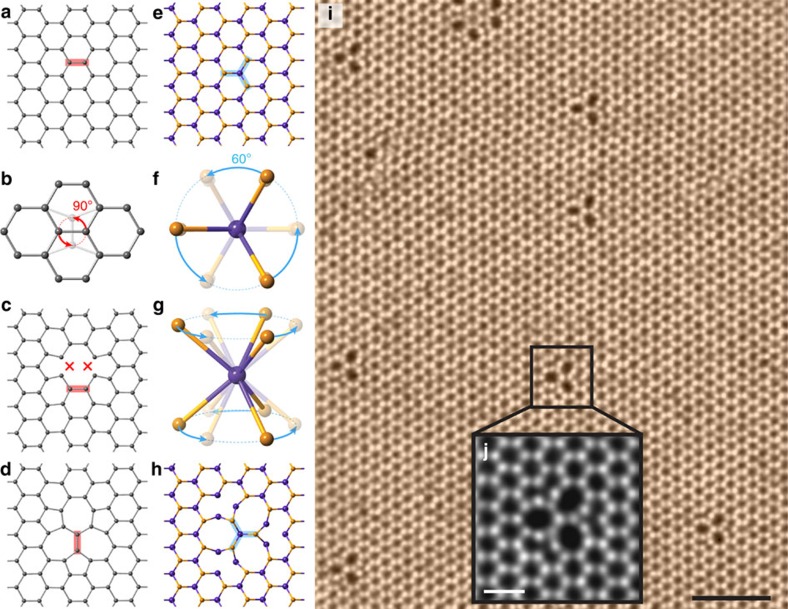
Model of rotational defect in graphene and TMDs. (**a**) Atomic model of graphene. (**b**) Top view of the Stone–Wales transformation, showing 90° rotation of a carbon bond. (**c**) Atomic model of graphene with a divacancy. (**d**) The atomic model of SW-transformed graphene divacancy. (**e**) Atomic model of TMDs with a structural formula MX_2_, the top view. The orange spheres represent chalcogen atoms, the blue ones the metal atoms. (**f**,**g**) Triple M–X bonds showing 60° rotation from the top and perspective views. (**h**) The atomic model of trefoil defect. Three M–X bond pairs marked by blue bars are 60° rotated from those in **e**. (**i**) A typical ADF image of WSe_2_ observed at 500 °C. The density of trefoil defect is found to be about 5.1%. Scale bar, 2 nm. (**j**) The magnified ADF image from the black square in **i**. Scale bar, 0.5 nm.

Figure 1i presents examples of trefoil defects in WSe_2_, which were found abundant at elevated temperatures. A comparison of the experimental STEM images with the simulated ones based on possible atomic structures (Supplementary Fig. 2) indicated that the system is Se deficient due to electron-beam irradiation. Similar trefoil-shaped defects also exist in graphene as reconstructed divacancies, but there the C–C bond is rotated by 90° as shown in Fig. 1c,d.

**Figure 2:**
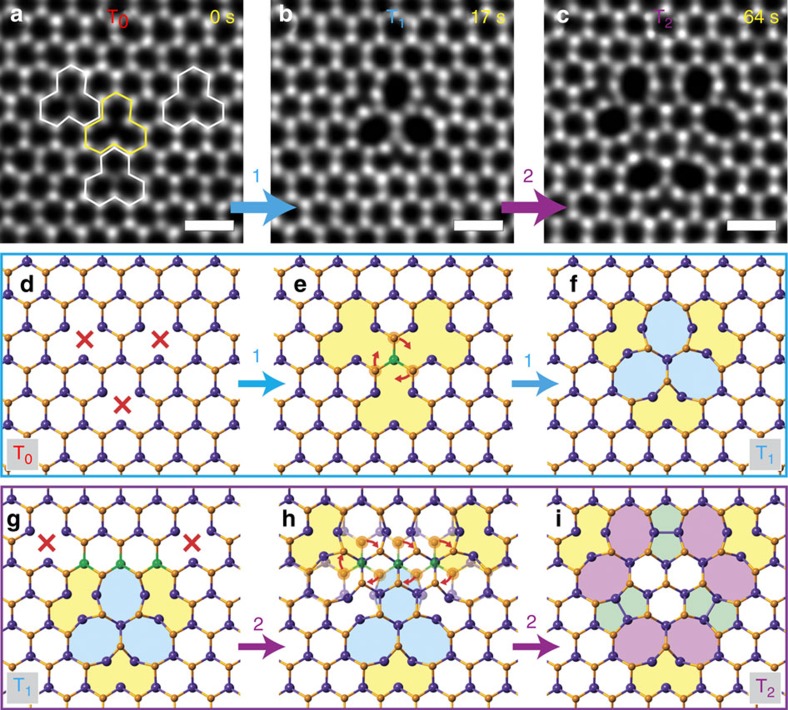
Structure, formation and evolution of rotational defect in WSe_2_. The filtered ADF images of WSe_2_ of (**a**) SV_Se_ (white polygon) and DV_Se_ (yellow polygon), (**b**) T_1_ defect and (**c**) T_2_ defect. (**d**–**f**) The atomic model of T_0_ to T_1_ transformation including the creation of three DV_Se_ (red crosses) (**d**) and a 60° rotation of three pairs of W–Se bonds around the W atom (green) **e** to form T_1_ with three octagons (blue; **f**). (**g**–**i**) The atomic model of T_1_ to T_2_ transformation. (**g**) Two DV_Se_ are created at the vicinity of T_1_ defect. (**h**) Seven pairs of W–Se bond rotations according to the W atoms marked by green color. (**i**) The T_2_ defect. Scale bar, 0.5 nm.

Figure 2a–c shows the filtered annular dark-field (ADF) images of three different Se-deficient WSe_2_ structures. In Fig. 2a, one can see two distinct image contrasts of the vacancies at the Se sites, corresponding to single Se vacancies (SV_Se_, white polygon) and double vacancies (DV_Se_, yellow polygon), respectively. At *t*=17 s, a new WSe_2_ defect of three-fold rotational symmetry, consisting of three eight-membered rings, resembling a trifoliate leaf, suddenly appears (Fig. 2b). We designate this three-fold symmetrical trefoil defect as the ‘first-order rotational defect’ or ‘T_1_’ (with the lattice keeping the original symmetry being ‘T_0_’). A model of the defect transformation from T_0_ to T_1_ is presented in Fig. 2d–f. The trefoil defects were always observed in W-centred configuration, but never in the equally possible Se-centred configuration, indicating the crucial role of the Se vacancies in the formation of the defect. We stress that the actual transformation mechanism may include not only bond rotations, but also the migration and rearrangements of Se atoms in the region with high vacancy concentration. By quantitative STEM simulations, the metal-centred T_1_ defect with 3DV included (T_1_(3DV)) is a best fit to the experimental results (Supplementary Figs 1 and 2 and Supplementary Note 1). We have also observed such defects in WS_2_ and MoSe_2_, but not in MoS_2_.

The trefoil defects persist when the samples are cooled to room temperature (Supplementary Fig. 3 and Supplementary Note 2). We also found that trefoil defects can be produced directly at room temperature. However, in the latter case, the defects usually appear in asymmetric shapes with only two octagon leaves as shown in Supplementary Figs 4,5 and 7, because the energy barriers for bond rotations are relatively high, and the multistep transformations may require longer times than at high temperatures.

To rationalize the experimental findings, we carried out first-principles calculations of the atomic structures and formation energies of the defect structures. We define the formation energy *E*_f_ of a defect in a TMD MX_2_ as


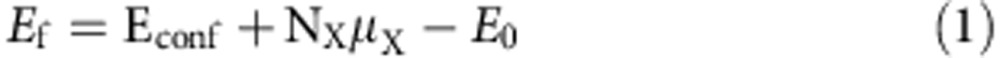


where *E*_conf_ is the energy of the supercell with the defect, *E*_0_ is the energy of the pristine supercell, *N*_X_ are the number of missing chalcogen atoms and *μ*_X_ their chemical potential with respect to X_2_ molecule. Our calculations show that rotating three W–Se bonds by 60° after 3DV_Se_ were created (Fig. 2e) lowers the energy of the system by 1.8 eV. Supplementary Table 1 lists the formation energies of the trefoil and various rotational defects in the S/Se-deficient MoS_2_, MoSe_2_, WS_2_ and WSe_2_ lattice, for convenience given as normalized per chalcogen vacancy. The T_1_(3DV) has lower formation energy per S/Se vacancy than SV or other defect structures and in particular the T_1_(3SV) defect is strongly unfavourable. For the MoS_2_, the staggered DV line structure^18^ shows lower energy than the T_1_(3DV) defect, which may explain that T_1_(MoS_2_) defect has not been found in our experiments.

### Expansion of trefoil defects by multiple M–X bond rotations

Figure 2c shows a larger second-order rotational defect (T_2_) in WSe_2_. The T_2_ defect has a triangular shape and is about twice the size of T_1_ with each edge containing two octagons (six octagons in total, purple) as shown in the model in Fig. 2i. A model of the atomic rearrangement during the transformation from T_1_ to T_2_ is presented in Fig. 2g–i. When two more DV_Se_ are created near the T_1_ (red crosses in Fig. 2g), the structure can be transformed into T_2_ by rotating seven pairs of W–Se bond (Fig. 2h) around the three W rotation centres (green atoms in Fig. 2g). The reverse transformation that restores, for example, T_1_/T_2_ to T_0_ by another set of M–X bond rotations was also observed (see Supplementary Movie 1). When the rotational defect transforms to a larger defect (for example, T_2_ to T_3_), a larger number of collective M–X bond rotations might require higher energy barriers, as a result, the transformation process is divided into more steps of M–X bond rotation (see Supplementary Figs 8 and 9, Supplementary Movie 2 and Supplementary Note 3).

### Migration of trefoil defects

**Figure 3:**
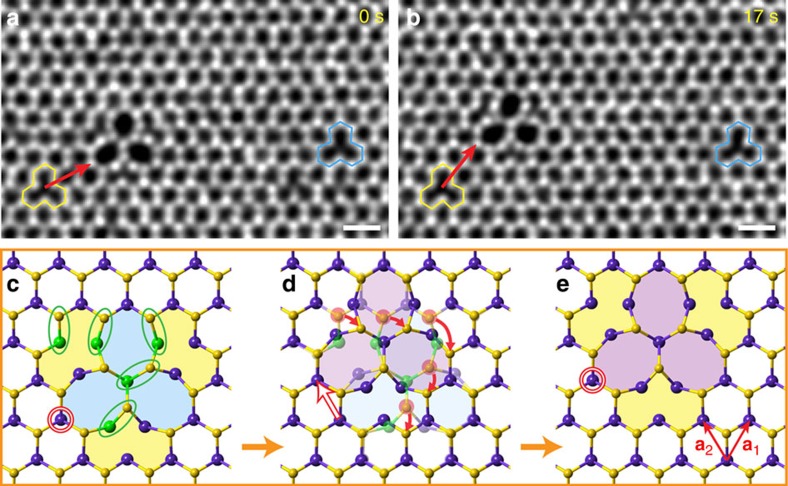
Migration of trefoil defect in TMDs. (**a**,**b**) T_1_ defect migration in WSe2. The sequential STEM images were carefully aligned according to the vicinal large cluster (not shown) using the ImageJ software. Two Se divacancies (yellow and blue polygons) are stable in the consecutive frames, and used as references. (**c**–**e**) The migration model of T_1_ defect glides a lattice constant distance towards the a_1_ direction. Scale bar, 0.5 nm.

During our STEM observations, not only the Se vacancies but also the trefoil defects were mobile (Supplementary Figs 6 and 10, Supplementary Movies 3 and 4 and Supplementary Note 4). The migration rate of the trefoil defects is much lower than that of SV or DV, presumably due to the collective rearrangement of a larger number of atoms. Figure 3a,b shows an example of in-plane gliding of T_1_(WSe_2_) by one lattice constant distance (see Supplementary Movie 5). Two Se divacancies, highlighted by yellow and blue polygons, were stable in the consecutive ADF images and acted as the reference. The initial and final locations of the trefoil defect are indicated by the red arrows that point to the corners of the octagons according to the yellow divacancy. Figure 3c–e shows an atomic model of T_1_ defect gliding. Five pairs of W–S bonds (marked by green circles in Fig. 3c) rotate collectively clockwise (red arrows in Fig. 3d) around five W rotation centres (green atoms) to accomplish the migration of the T_1_ defect in a distance equal to one lattice constant in the **a**_**2**_ direction (red hollow arrow). Larger T_2_(WSe_2_) defects can also migrate in the host lattice (Supplementary Fig. 11, and Supplementary Movie 6) by a similar mechanism, with multiple M–X bond rotations.

### p-type doping and magnetism of large trefoil defect and 8–5–5–8 domain boundary

**Figure 4:**
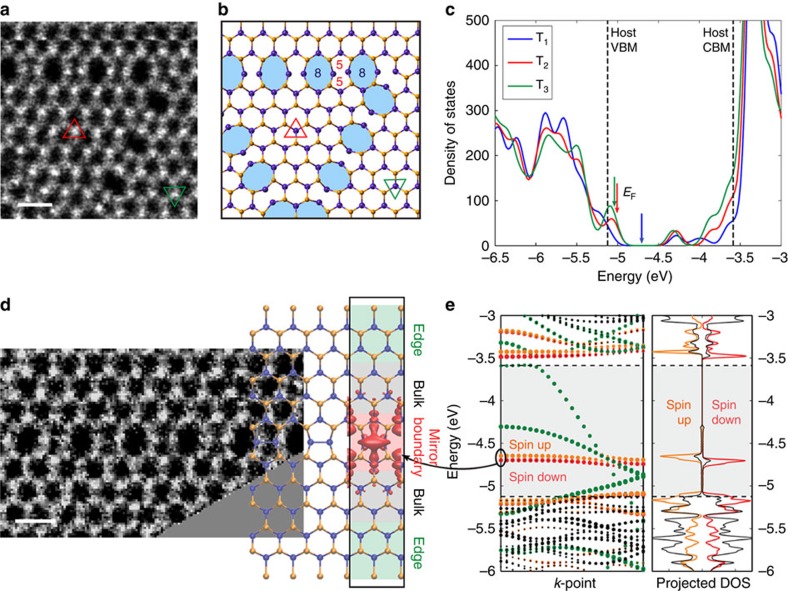
Large trefoil defect and magnetic 8–5–5–8 (δ) domain boundary. (**a**) The ADF image of T_4_(WSe_2_). (**b**) The corresponding atomic model of T_4_(WSe_2_) shown in **a**. Larger rotational defect keep the triangular shape and include three boundaries consisting of 8–5–5–8 membered rings (δ boundary). The orientation of the inner domain of the T4 defect is 60° (or 180°) rotated from the outer domain. (**c**) Calculated density of states from T_1_ to T_3_ defects. The Fermi-level positions are denoted with arrows. (**d**) The ADF image of the boundary from T_6_(WSe_2_) with the atomic structure partially overlaid. (**e**) The edge band structure and density of states of the δ boundary with colours denoting projections to different regions within the ribbon geometry used in the calculation. Dashed lines denote the band edge positions of pristine WSe_2_. The wavefunction isosurface for the mid-gap state localized to the δ boundary is also shown in **b**. Energy zero is at the vacuum. Scale bar, 0.5 nm.

If the M–X bonds rotate further, the rotational defects can expand to even larger sizes. Figure 4a shows an ADF image of T_4_(WSe_2_) defect. According to the structural parameters for rotational defects listed in Table 1, the T_4_ defect involves 36 pairs of M–X bond rotation around 16 W rotation centres, which leads to a large triangular shape and each edge contains four octagons sandwiched with a pair of pentagons, as illustrated in Fig. 4b. Note that the 8–5–5–8 edges form linear domain boundaries (δ boundary), which are further visualized in Fig. 4d (extracted from the T_6_ defect). The calculated density of states from T_1_, T_2_, and T_3_ defects in WSe_2_ are shown in Fig. 4c. The additional states in the mid gap originate from the corners of the defect, whereas the states close to the valence band maximum and conduction band minimum can be traced to the edges (see Supplementary Figs 12 and 13). Interestingly, as the size of the defect increases, the Fermi-level moves closer to the valence band maximum, that is, the edge states of middle-sized defects essentially lead to p-type doping of the system. On the other hand, recent tight-binding transport calculations showed that the conductivity of MoS_2_ sheets with large rotational defects and grain boundaries containing 8–5–5–8 rings is strongly reduced across such defects^21^. We did not observe any rotational defects in MoS_2_, but their effects on the electronic transport in other TMDs we studied may be similar.

Figure 4e shows the electronic structure and the local density of states of the ideal δ boundary in WSe_2_. The electronic states localized to the boundary have now energies in the lower mid-gap region with very small dispersion and consequently large density of states. The conductivity along these boundaries will then strongly depend on the Fermi-level position within the gap. These states were also found to carry a magnetic moment. A similar behaviour was reported for the 5–7 dislocation^22^ and 8–5–5–8 boundary in MoS_2_ (ref. 23) and epitaxial graphene on Ni(111)^24^.

## Discussion

Calculating the elastic properties of pristine and defective WSe_2_, we found that a Se vacancy concentration of 3%, in the range of the experimentally observed concentrations, decreases the bulk modulus by only about 4%. This indicates that inducing these defect structures only weakly deteriorates the mechanical properties of the system while giving rise to substantial changes in the electronic structure.

As shown in the present study by direct visualization of atomic-scale transformations, TMDs feature a rich variety of rotational defects, with the structures and their formation mechanisms related to the symmetry of the lattice but clearly distinct from those in other 2D materials such as graphene and h-BN. Phase transitions^25^ and inverse domains may be induced a posteriori, for example, by electron-beam irradiation, which leads to drastic changes in the material properties, for example, p-type doping and magnetism. The bond rotation mechanism reported here not only gives rise to a new class of defects, but also adds to the toolbox of available means for modifying the local properties of TMDs.

## Methods

### Material synthesis and sample preparation

Single crystal of MoS_2_, MoSe_2_, WS_2_ and WSe_2_ were grown by chemical vapour transport method using either Br_2_ as a transport agent at 950 °C. Ten grams of 99.99% purity of Mo or W, and S or Se elements with 5 mg cm^−3^ of Br_2_ were cooled in a quartz tube ampoule with liquid nitrogen and sealed in vacuum (~1 × 10^−6^ Torr)^26^,^27^. Single-layer TMDs were mechanically exfoliated from synthesized crystals using Scotch tape and transferred to 300 nm SiO_2_/Si substrate. To grow WSe_2_ by chemical vapour deposition, WO_3_ and Se powders were, respectively, used as the tungsten and selenium sources, which were placed in two separate quartz boats located in upstream of a gas flow. The temperatures of the WO_3_ and Se powders were set at 850 and 250 °C, respectively. The sapphire substrates where the WSe_2_ flakes were deposited at 750 °C were placed at the downstream side with WO_3_ and Se vapours being transported by an Ar/H_2_ flowing gas (Ar=60 s.c.c.m. and H_2_=3 s.c.c.m.). During the growth, the pressure of reaction chamber keeps constantly at 70 Torr. The specimens were transferred to TEM microgrid and heated in the TEM chamber (vacuum level of ~1.8 × 10^−5^ Pa) at 500 °C in a JEOL heating holder.

### STEM-ADF-imaging experiments

STEM-ADF imaging was performed using an aberration-corrected JEOL-2100 F equipped with a DELTA corrector and cold field-emission gun. The microscope was operated at an accelerating voltage of 60 kV. The convergence semi-angle was set to 35 mrad, and the inner acquisition semi-angle was 79 mrad. The probe current was 10–15 pA. The sequential ADF images were recorded with 512 × 512 pixels and acquired with 16–64 μs dwell time. The ADF images in Figs 1, 2, 3 have been processed by low-pass filter to enhance the contrast. False-colour images and the image alignment were processed using ImageJ.

### Computational details

Density functional theory calculations were carried out with the PAW method as implemented in the VASP software package^28^,^29^, using the PBE exchange-correlation functional^30^. The defects were modelled in periodically repeated 10 × 10 supercells of the primitive trigonal lattice with the sheets separated by 16 Å of vacuum. A single k-point was used for BZ integration and the plane-wave basis set cutoff was 300 eV. For a few selected structures, the supercell size was increased to 14 × 14 and the energy cutoff increased to 400 eV, which produced an error of at most 0.1 eV, which is our estimated numerical accuracy. Calculations for ideal boundaries were carried out in the ribbon geometry with five MX_2_ units on each side of the boundary and with fully chalcogen passivated edge structures, as shown in Fig. 4d. Twelve k-points along the ribbon were found to yield converged results. Robustness of the magnetization was checked by performing additional calculations with 24 k-points and of varying the smearing parameter. The results for MoSe_2_ look very similar to the WSe_2_ results.

## Author contributions

F.-S.H., K.-H.L., J.J. and Y.-S.H. synthesized the MoS_2_, MoSe_2_, WS_2_, WSe_2_ single crystals by the CVT method. P.-Y.T., C.-H.Y. and P.-W.C. synthesized the single-layer WSe_2_ by the CVD method. Y.-C.L. and K.S. planned the project. Y.-C.L. performed the experiments and analysed the STEM data. T.B., H.-P.K. and A.V.K. performed the DFT calculations. Y.-C.L., T.B., H.-P.K., A.V.K. and K.S. co-wrote the paper. All authors discussed the results and comments on the manuscript.

## Additional information

**How to cite this article**: Lin, Y-C. *et al*. Three-fold rotational defects in two-dimensional transition metal dichalcogenides. *Nat. Commun.* 6:6736 doi: 10.1038/ncomms7736 (2015).

## Supplementary Material

Supplementary Figures, Supplementary Table, Supplementary Notes and Supplementary Reference Supplementary Figures 1-13, Supplementary Table 1, Supplementary Notes 1-4 and Supplementary Reference

Supplementary Movie 1 Expansion and restoration of a trefoil defect, T_0_ to T_1_ to T_2_ to T_0_ at T=500°C.

Supplementary Movie 2 Expansion of a trefoil defect, T_2_ to T_3_ at T=500°C.

Supplementary Movie 3 Migration of S vacancies in MoS_2_ at T=500°C.

Supplementary Movie 4 Migration of Se vacancies in WS_e2_ at T=500°C.

Supplementary Movie 5 Migration of a T_1_ trefoil defect in WS_2_ at T=500°C.

Supplementary Movie 6 Migration of a T_2_ trefoil defect in WS_e2_ at T=500°C.

## Figures and Tables

**Table 1:** Structural parameters for trefoil-like defects.

	T_1_	T_2_	T_3_	T_*n*_
Rotation centre (*R*_c_) for expansion	1	4	9	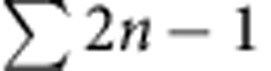
Pairs of M–X bond rotation (*R*_P_)	3	10	21	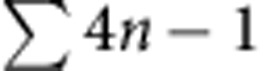
Octagons	3	6	9	3*n*
Pentagons	0	6	12	6(*n*-1)
DV(S/Se)	3	5	7	(2*n*+1)
Defect size (*A*_*n*_)	12*A*_0_	25*A*_0_	42*A*_0_	(2*n*^2^+7*n*+3)*A*_0_
*R*_c_ and *R*_P_ for migrating a lattice constant	5	9	13	4*n*+1

Unit cell size *A*_0_=9.37 Å^2^.
