# Supplementary material for: Three-fold rotational defects in two-dimensional transition metal dichalcogenides
Source: Nat Commun. 2015 Apr 2;6:6736. doi: 10.1038/ncomms7736 (PMC4396367; doi:10.1038/ncomms7736)
Supplement: Supplementary Figures, Supplementary Table, Supplementary Notes and Supplementary Reference — Supplementary Figures 1-13, Supplementary Table 1, Supplementary Notes 1-4 and Supplementary Reference [file ncomms7736-s1.pdf]

Supplementary Figures

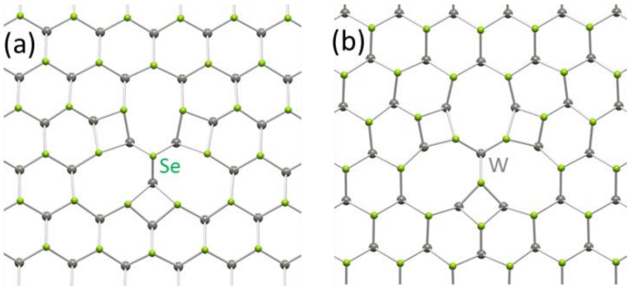

Supplementary Figure 1. DFT calculation of the 60° rotation defect. (a) Se centered and **(b)** W centered defect without Se atom loss. The formation energies are 6.9 eV and 10.7 eV, respectively. Green and grey spheres represent Se and W atoms, respectively.

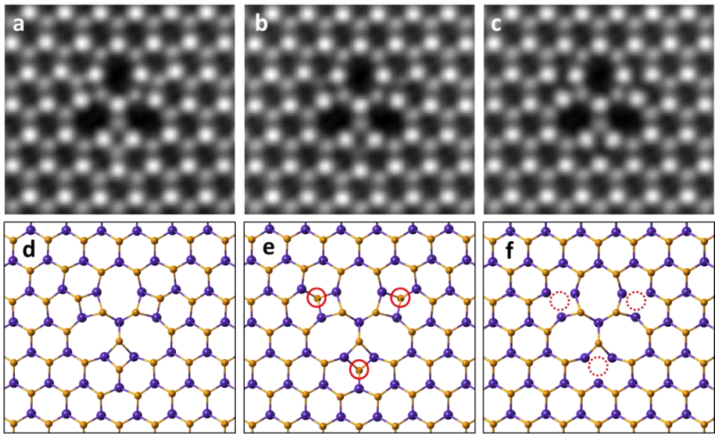

Supplementary Figure 2. Atomic structure and STEM simulation of T_1 defect. (a)-(c) The STEM simulation images of W centered $T_1(WSe_2)$ defect, where the corresponding atomic models are shown in **(d)-(f)**. **(a,d)** T_1 defect without Se atom loss, **(b,e)** T_1 defect with 3 Se single vacancies highlighted by red circles, **(c,f)** T_1 defect with 3 Se divancies highlighted by red dotted circles. The image simulation is made by QSTEM.

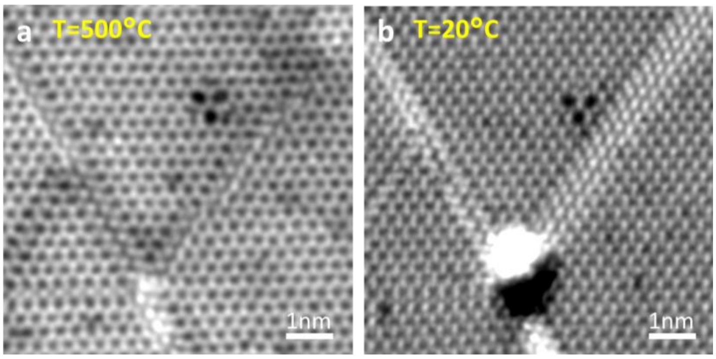

Supplementary Figure 3. Observations of a T1 defect at room temperature. The STEM images of **(a)** trefoil defect created at 500°C and **(b)** the trefoil defect retained at the same position after cooling down to room temperature. The time interval for cooling and sample stabilizing is 2hr.

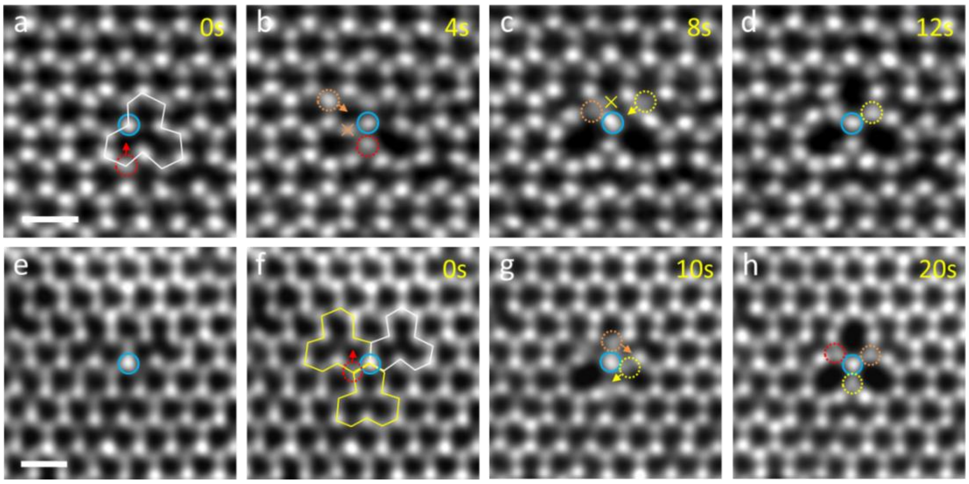

Supplementary Figure 4. T_1 defect created at room temperature. (a) The Se divacancy marked by a white polygon existed in the vicinity of the W atom marked by the blue circle. The Se atom pair (red dotted circle) hopped a half lattice constant toward the red arrow. **(b)** At t = 4s, one Se atom pair were removed by e-beam (orange cross) and another Se atom pair (orange dotted circle) hopped toward the orange arrow. **(c)** At t = 8s, one Se atom pair were removed by e-beam (yellow cross) and another Se atom pair (yellow dotted circle) hopped toward the yellow arrow to form the symmetrical trefoil defect as shown in **(d)**. **(e),(f)** The identical ADF image of WSe2 at room temperature. The metal rotation center is marked by blue circle. Two single Se vacancies and two divacancies are marked by yellow and white polygons, respectively. **(f)** The Se atom pair (red dotted circle) rotate 60° toward the red arrow. **(g)** The other two Se atoms (orange and yellow dotted circles) also rotate 60° to form trefoil defect **(h)**. Scale bar: 0.5 nm.

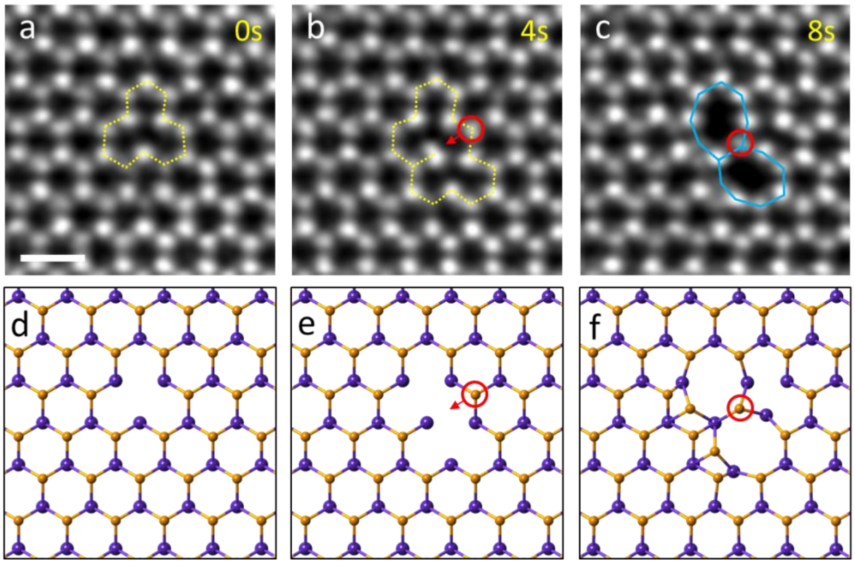

Supplementary Figure 5. Defect with two octagon leaves. (a) One Se divacancy was created. **(b)** Two nearest neighbor Se divacancies were formed and a Se atom pair hopped toward the red arrow to form **(c)** a defect with two octagon leaves. **(d)-(f)** The corresponding atomic model. Scale bar: 0.5 nm.

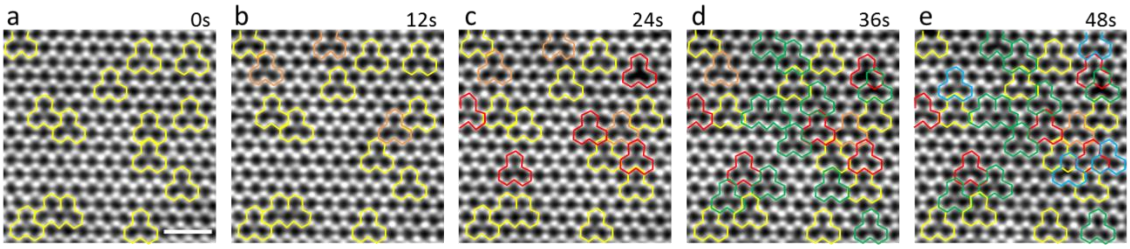

Supplementary Figure 6. Migration of Se vacancies at RT. (a)-(e) Consecutive STEM image of WSe_2 at RT. The Se single or divacancies were marked with color polygons. The new created or position changed Se defects were displayed in different color, i.e., **(b)** orange, **(c)** red, **(d)** green, **(e)** blue. **(e)** After t = 48s, there were about 70% of Se vacancies stayed fixed (yellow polygons). Scale bar: 1 nm.

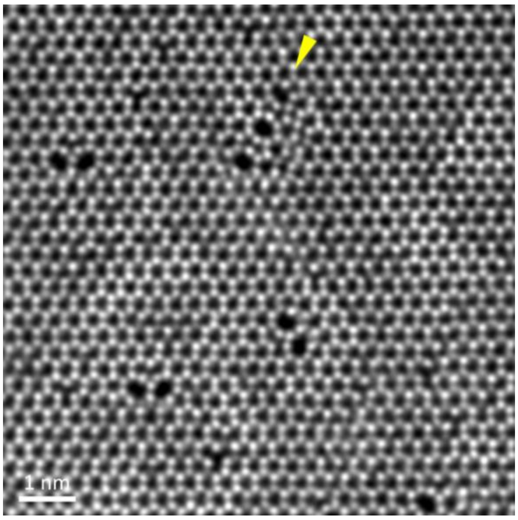

Supplementary Figure 7. Defect with two octagon leaves and linear defect created at room temperature. The STEM image of WSe_2. The short linear 8-5-5-8 defect is pointed by yellow arrow.

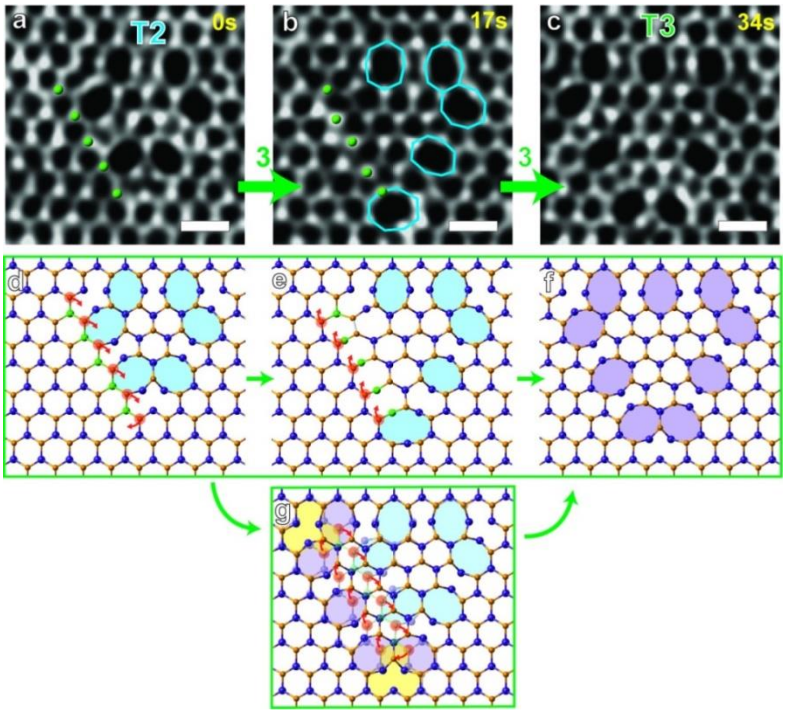

Supplementary Figure 8. Multistep transformation process in large rotational defect. (a)-(c) Sequential ADF images of WSe_2 showing T_2 to T_3 transformation. **(d)-(f)** The corresponding atomic model for T_2 to T_3 transformation by sequential W-Se bond rotation. Five W rotation centers are highlighted by five green atoms. **(d)** Half of the Se atoms around the rotation centers (6 pairs of Se atoms on the right hand side marked in red) rotate first and forms the half-transformed intermediate structure **(b)**. Some Se atoms in the intermediate structure might overlapped with the W atoms and show brighter contrast. **(e)** The other half of the Se atoms around the rotation centers (5 pairs of Se atoms on the left hand side) rotate again to complete the transformation. **(g)** The atomic model for the transformation by rotating 11 pairs of W-Se bond collectively. Scale bar 0.5 nm.

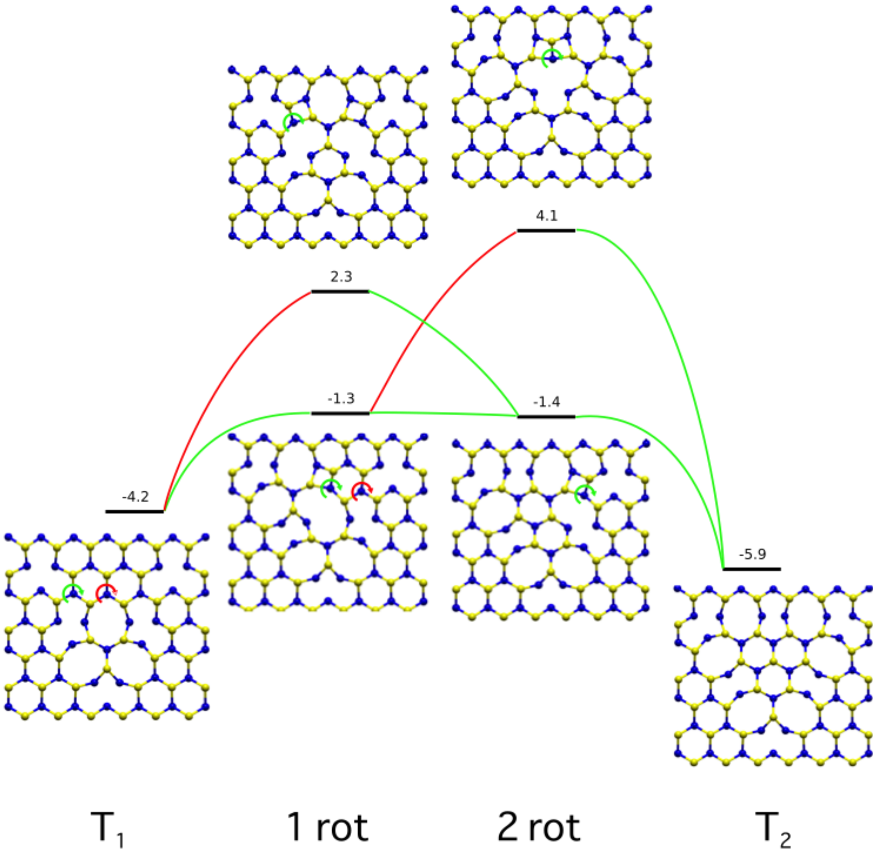

Supplementary Figure 9. T_1 to T_2 transformation by successive bond rotations. Energies of individual configurations from T_1 to T_2 in WSe_2 (Fig. 2g-i of the main paper) when rotating individual W centered units by 60˚. A lattice with ten single Se vacancies is taken as the zero of energy.

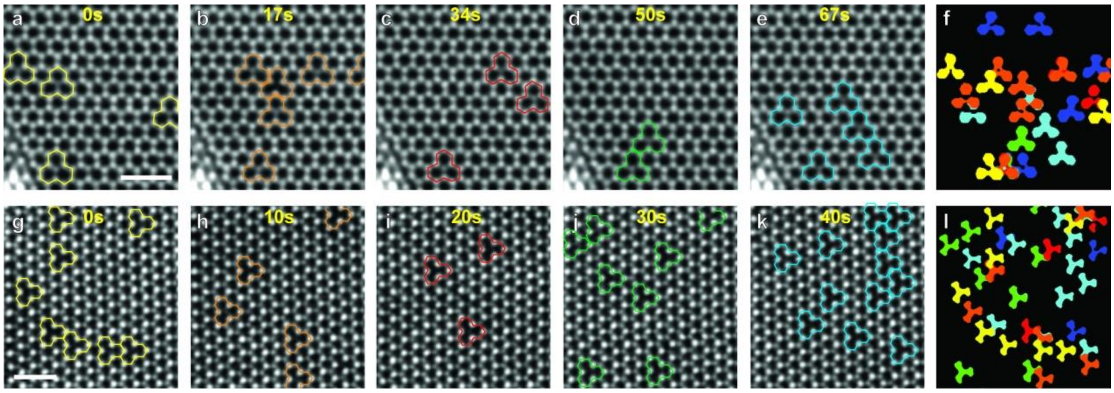

Supplementary Figure 10. Migration of single and double S/Se vacancies in TMDs. (a)-(e) Consecutive ADF images of WSe_2. **(g)-(k)** Consecutive ADF images of MoS_2. The $SV_{S/Se}$ and $DV_{S/Se}$ are marked by color polygons and the corresponding S/Se vacancies mappings are shown in **(f)** and **(l)**. The vacancy mappings were processed by ImageJ. Scale bar 1 nm.

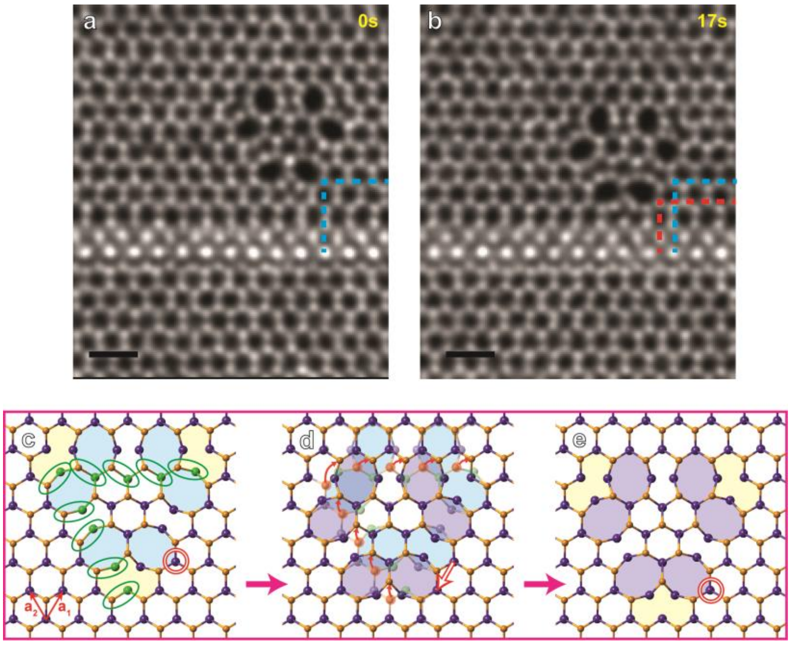

Supplementary Figure 11. Migration behavior of T_2 defect. (a,b) Sequential ADF image of $T_2(WSe_2)$. The blue and red dashed lines indicate the positions of the T2 defect. **(c)-(e)** The migration model of T_2 defect which glide a lattice constant distance toward the **–$\mathbf{a_1}$** direction. Scale bar 0.5 nm.

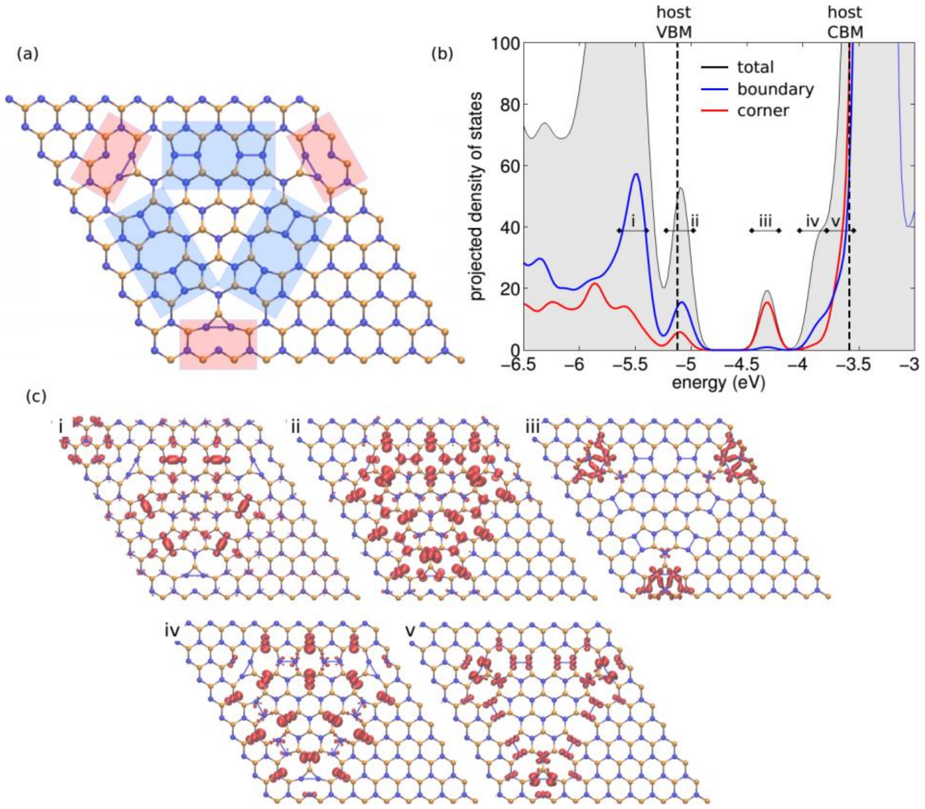

Supplementary Figure 12. Electronic structure of the trefoil defects. (a) The atomic structure of T_3 defect. **(b)** Projected density of states from all W atoms (black), from W atoms at the boundary regions (blue), and from W atoms at the corner regions (red). These regions are explicitly denoted in the atomic structure **(a)**. **(c)** Isosurfaces for the partial charge densities related to each of the main peaks in **(b)**. The peak (iii) in the mid gap originates purely from the corners of the T_3 defect. There are also states close to the valence band maximum and conduction band minimum that have considerable boundary contributions, although mixed with other corner-localized and host-derived states. These can be compare to the pure boundary DOS shown in Fig. 4(b,c), which showed two states close to VBM (one within the gap) and one state close to CBM. In particular, the isosurface shown in Fig. 4(d) shows features that are similar to both the (i) and (ii) peaks and thus it likely derives from both of these states.

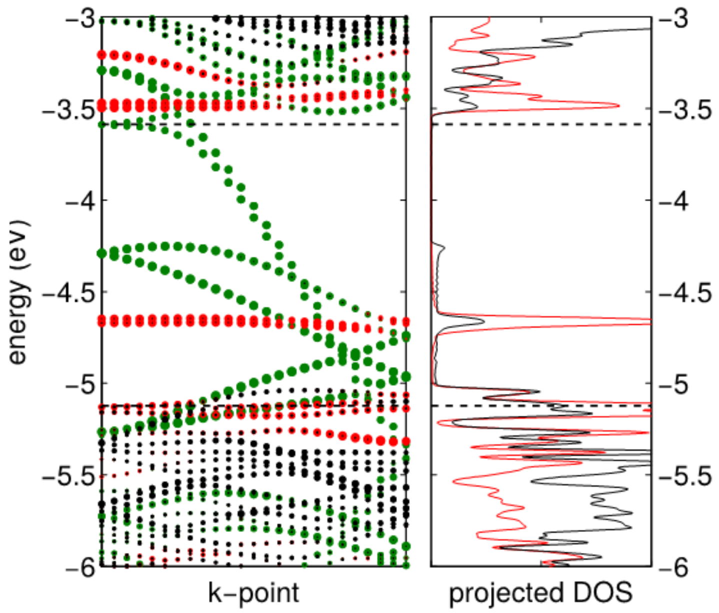

Supplementary Figure 13. The band structure and density of states of the 8-5-5-8 domain boundary calculated with spin-orbit coupling. The colors and notations are the same as in Fig. 4e of the main paper. For the states localized at the domain boundary (red), the effect of spin-orbit is minimal, both for position of the states within the gap as well as splitting of the states. The states localized at the edges of the ribbon (green) undergo more significant splitting, but otherwise show the same general features.

Supplementary Table 1 | Formation energies of defects of the S/Se-deficient lattice

**Material**	**Formation energy normalized per S/Se vacancy (eV per S/Se vac.)**				
	**SV**	**DV**	**T_1(3SV)**	**T_1(3DV)**	**Stg-DV line**
**MoS_2**	3.45	3.43	5.12	3.35	3.28
**$MoSe_2$**	3.39	3.23	4.55	2.93	3.08
**WS_2**	3.56	3.46	5.30	3.42	3.48
**WSe_2**	3.42	3.16	5.06	2.86	3.20

Supplementary Note 1. Atomic structure of trefoil defects.

The trefoil defects were always experimentally observed in W-centered configuration, but never in the Se-centered configuration. Indeed, the calculated formation energies of the W-centered or Se-centered trefoil defect with no Se atom loss are 10.7 and 6.9 eV (Supplementary Fig. 1), making the Se-centered defect preferable in the pristine lattice. Even though the formation energies of other defects are within the range of what has been seen for Stone-Wales transformations in graphene and 2D-SiO_2 [1]. By comparing with STEM simulations as shown in Supplementary Fig. 2, the W-centered trefoil defect ($T_1(WSe_2)$) including with 3 Se divacancies (Supplementary Fig. 2c,f) shows the best fit to the experimental results (Fig. 2b).

In our experience, the trefoil defects can be created in WS_2, $MoSe_2$, and WSe_2 by electron beam at elevated temperatures, but have never been observed in MoS_2. On the contrary, the phase transition (2H to 1T) much easily happens in MoS_2 under the electron beam, but hardly happens in group 6 metal diselenides. We emphasize that different materials can have different phases or defects. The group 6 metal diselenides were found to be more stable in the 2H phase and exhibit more frequent bond rotations to form trefoil defect.

Supplementary Note 2. Trefoil defects created at room temperature.

The trefoil defects can be observed also at room temperature (RT). Supplementary Figure 3 shows a T1 defect, retained in the WSe2 lattice during the 500°C heating (a) and cooling (b) cycle within the time interval about 2 hrs. Although such a rapid cooling may cause some local structure transformations or sample damage, some of the trefoil defect still retained their original positions, which further demonstrates the stability of trefoil defects.

Supplementary Figure 4a-4d shows consecutive STEM images during the creation of a T_1 defect at RT. The formation mechanism involves Se atoms hopping a half lattice constant, which is different from the M-X bond rotation, because the bond rotation requires relatively higher energy than the energy of Se atoms hopping. A similar transformation can be found (Supplementary Fig. 4e-4h) when three Se single- or divacancies are in a certain arrangement as shown in Supplementary Fig. 4f. This multi-step transformation process can be recorded due to the relatively higher energy barrier for bond rotation at room temperature (Supplementary Fig. 4g-4h), which slows the process down.

Supplementary Figure 5 shows details of the formation mechanism and the corresponding atomic model. Beside this, we found that the chalcogen vacancies are less mobile at RT (Supplementary Fig. 6) than at elevated temperature (Supplementary Fig. 10), which may influence the RT formation of the trefoil defect. We found that the defects created at RT are mostly asymmetrical and form a shape of two octagon leaves as shown in Supplementary Fig. 7. Not only the two-leaved defect, but also a short linear 8-5-5-8 defect can be created by electron beam at RT. Note that it is more difficult to create the larger symmetrical trefoil defects at RT than at elevated temperature due to the requirement of more steps in the transformation. It is hard to ascertain precisely how many Se vacancies are involved in the structure of Supplementary Fig. 5f, but calculations reveal that all possible configurations have formation energies of 5.8-6.8 eV per Se vacancy, which shows that these are metastable structures that would eventually form the T_1 defect given enough time.

Supplementary Note 3. Expansion to large rotational defect by multiple bond rotations.

We have calculated the formation energies of a series of intermediate structures from T_1 to T_2 formed by 60° rotations of individual bonds (see Supplementary Fig. 9) and found that the formation energies are rather high. Since these energies would be an underestimate of the transformation barrier, from the observed high speed of the transformation we conclude that the rotational defects are created by a collective M-X bond rotation.

Supplementary Note 4. Migration of vacancies and trefoil defects in TMDs.

Supplementary Figure 10a-f and 10g-l show two example of chalcogen vacancy migration in two consecutive images in WSe_2 and MoS_2, respectively (See also Supplementary Movie 3 and 4). The migration barrier of SV_S to the nearest S site is about 2.3 eV, and it can easily be overcome under the e-beam irradiation at elevated temperatures. Although we cannot trace every atom or vacancy and assign its diffusion pathway, over 90% of the S and Se vacancies in MoS_2 and WSe_2 were found to change position during sequential STEM imaging, and they appear to be distributed randomly in the lattice, neither segregation nor any ordered structures can be found.

Supplementary References

1. Björkman, T. *et al.* Defects in bilayer silica and graphene: common trends in diverse hexagonal two-dimensional systems. *Sci. Rep.* **3:3482**, 1-7 (2013).
