# Supplementary figures and images for: Three-fold rotational defects in two-dimensional transition metal dichalcogenides
Source: Nat Commun. 2015 Apr 2;6:6736. doi: 10.1038/ncomms7736 (PMC4396367; doi:10.1038/ncomms7736)

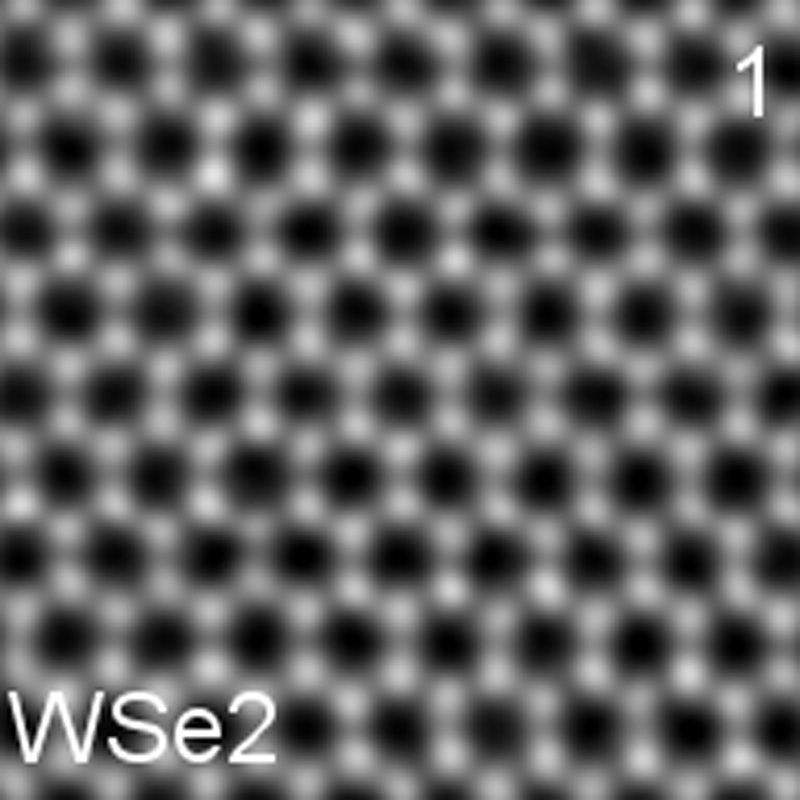

Supplement: Supplementary Movie 1 — Expansion and restoration of a trefoil defect, T0 to T1 to T2 to T0 at T=500°C. [file ncomms7736-s2.tif]

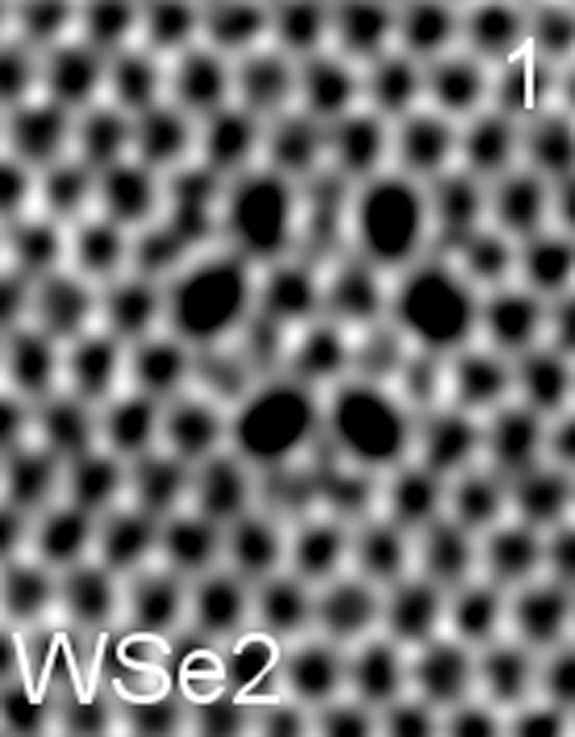

Supplement: Supplementary Movie 2 — Expansion of a trefoil defect, T2 to T3 at T=500°C. [file ncomms7736-s3.tif]

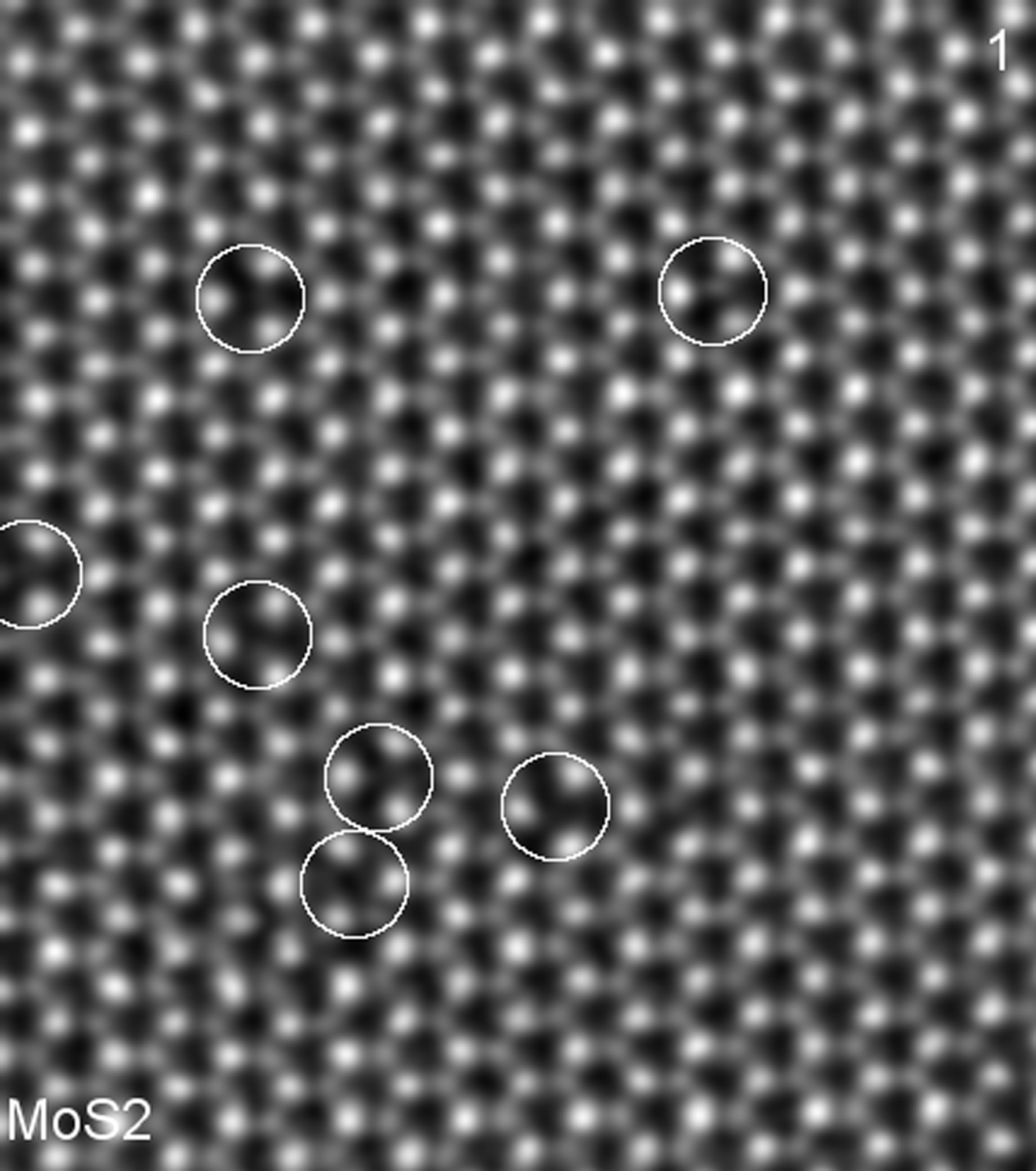

Supplement: Supplementary Movie 3 — Migration of S vacancies in MoS2 at T=500°C. [file ncomms7736-s4.tif]

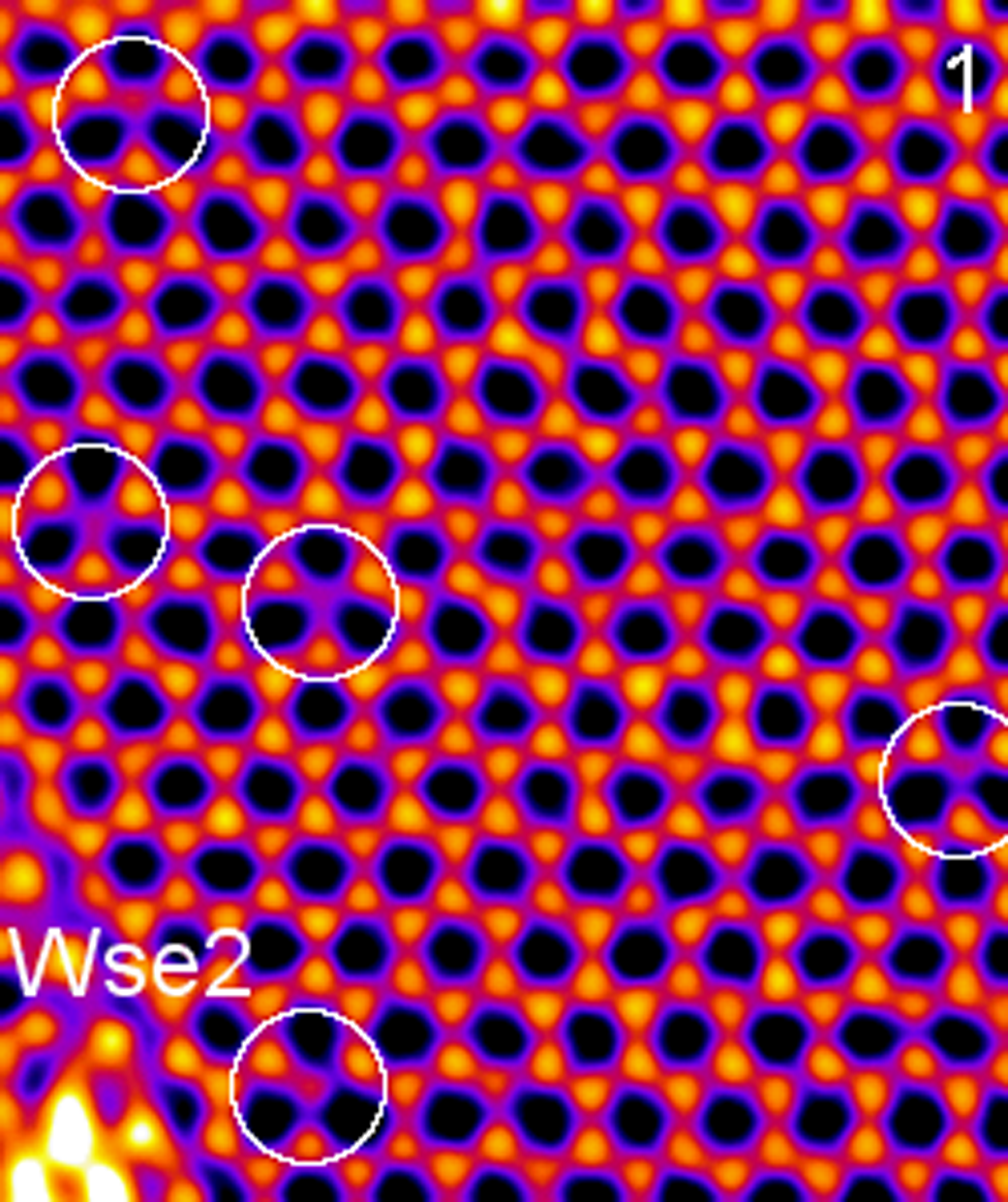

Supplement: Supplementary Movie 4 — Migration of Se vacancies in WSe2 at T=500°C. [file ncomms7736-s5.tif]

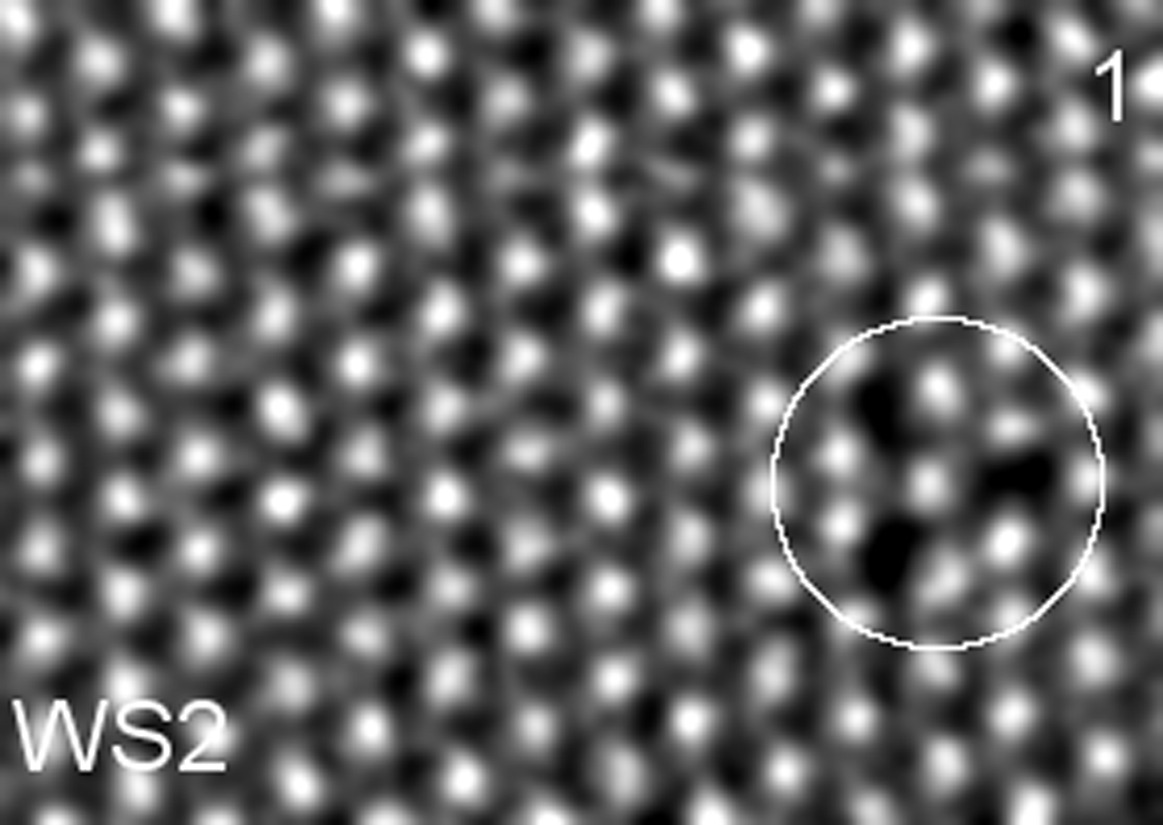

Supplement: Supplementary Movie 5 — Migration of a T1 trefoil defect in WS2 at T=500°C. [file ncomms7736-s6.tif]

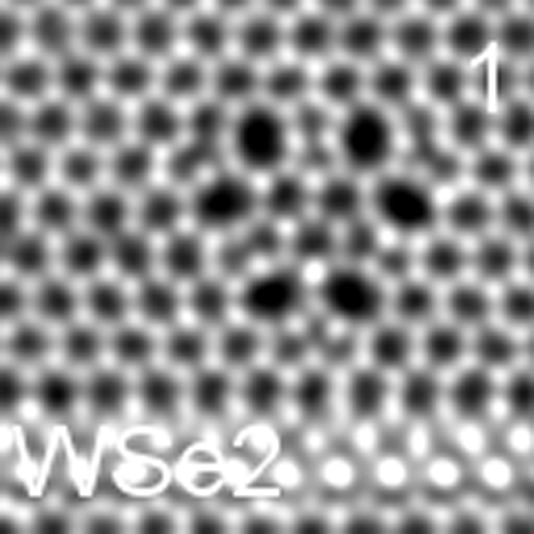

Supplement: Supplementary Movie 6 — Migration of a T2 trefoil defect in WSe2 at T=500°C. [file ncomms7736-s7.tif]
